# Factors affecting survival prognosis of patients with rectal cancer after neoadjuvant chemoradiotherapy

**DOI:** 10.3389/fonc.2025.1562634

**Published:** 2025-05-15

**Authors:** Baokun Li, Jiachao Han, Feifei Wang, Bin Yu, Guiying Wang, Fei Yang

**Affiliations:** ^1^ The Second Department of Surgery, The Fourth Hospital of Hebei Medical University, Shijiazhuang, Hebei, China; ^2^ Department of General Surgery II, Handan Central Hospital, Handan, Hebei, China; ^3^ Department of General Surgery, The Second Hospital of Hebei Medical University, Shijiazhuang, Hebei, China; ^4^ Department of Otolaryngology-Head and Neck Surgery, The Fourth Hospital of Hebei Medical University, Shijiazhuang, Hebei, China

**Keywords:** rectal cancer, locally advanced stage, neoadjuvant therapy, pathological complete response, prognosis

## Abstract

**Objective:**

To identify potential factors influencing the survival prognosis of locally advanced rectal cancer patients receiving neoadjuvant chemoradiotherapy.

**Methods:**

A retrospective study was conducted to collect data from January 2009 to December 2020 on 270 patients with locally advanced rectal cancer who were admitted to the Fourth Hospital of Hebei Medical University. The clinical data of patients before and after neoadjuvant chemoradiotherapy and postoperative treatment were compiled. The endpoints of the study were disease-free survival and overall survival of the patients. The univariate and multivariable regression analysis were used to identify factors that influence the patients’ survival prognosis.

**Results:**

Univariate analysis showed that factors associated with good prognosis in neoadjuvant chemotherapy patients included age <65 years, CEA value ≤5ng/mL, lymphocyte count >1.5×10^9^/L, normal albumin level, NLR ≤2.64, SII ≤683.16, PNI >49.23, tumor distance from the anal margin >5cm, tumor length ≤5cm, tumor invasion of the bowel wall ratio ≤50%, lower T stage and N stage, good tumor regression response, absence of KRAS gene mutation, and mismatch repair protein deficiency. And multivariate analysis showed that age (HR=0.385, P=0.007), NLR (HR=0.294, P=0.011), cT stage (HR=0.287, P<0.001), and tumor regression grade (HR=0.273, P<0.001) were significant factors influencing DFS in patients receiving neoadjuvant chemoradiotherapy. For OS, age (HR=0.497, P=0.035), cT stage (HR=0.387, P=0.001), and tumor regression grade (HR=0.307, P<0.001) were significant factors influencing OS in patients receiving neoadjuvant chemoradiotherapy.

**Conclusion:**

Age, cT stage, NLR, and tumor regression grade are significant factors influencing DFS and OS in patients with locally advanced rectal cancer. Younger age, lower cT stage, lower NLR value, and lower tumor regression grade are associated with better survival prognosis.

## 1 Introduction

Colorectal cancer is a global malignant disease with increasing incidence and mortality rates due to improving standards of living. According to the latest global cancer data in 2020, colorectal cancer ranks third in the incidence rate and second in the mortality rate among malignant tumors worldwide (1). In China, the incidence and mortality rates of colorectal cancer are slightly lower than the world average due to age structure, economic factors, and societal differences. However, both the incidence and mortality rates have been steadily increasing over the years, ranking fourth and fifth, respectively, among all malignant tumors in the country (2). Unlike in Western countries, rectal cancer is more common than colon cancer in China, accounting for over half of all colorectal cancers. Among rectal cancers, middle and lower rectal cancers are the most prevalent, with a higher proportion being in the advanced stages (3).

The fixed position of the rectum compared to the colon and the limited pelvic space, coupled with the influence of pelvic organs, make surgical treatment of locally advanced rectal cancer challenging and result in suboptimal curative outcomes. The National Comprehensive Cancer Network (NCCN) guidelines recommend neoadjuvant chemoradiotherapy (nCRT) for resectable locally advanced (stage II or III) rectal cancer and mandate nCRT for T4 or locally advanced unresectable rectal cancer (4). Neoadjuvant therapy, which includes radiation and chemotherapy, is administered before surgery and aims to reduce tumor volume, decrease lymph node enlargement, lower tumor staging, improve the rate of R0 resection, and achieve curative outcomes after surgery. According to the Chinese Society of Clinical Oncology (CSCO) guidelines, concurrent chemoradiotherapy is the recommended regimen for nCRT, with specific doses of radiation ranging from 45.0 to 50.4 Gy (1.8-2.0 Gy per fraction, 5 fractions per week, totaling 25–28 fractions), along with fluorouracil or capecitabine chemotherapy. Within the group of locally advanced rectal cancer (LARC) patients, neoadjuvant therapy can achieve pathological complete response (pCR) in approximately 8% to 35% of cases (5). Patients who achieve pCR have significantly better overall survival (OS) and disease-free survival (DFS) outcomes compared to those who do not achieve pCR (6). For patients with ultra-low rectal cancer, abdominal perineal resection or organ function-reducing surgery poses significant challenges to their quality of life and mental well-being. Neoadjuvant therapy increases the likelihood of sphincter preservation. For patients who achieve clinical complete response (cCR) after neoadjuvant therapy, an observation and wait-and-see approach can be adopted, as studies have shown no significant differences in 2-year DFS and OS between patients on observation and wait-and-see therapy versus those who undergo total mesorectal excision (TME) surgery (7). This approach also reduces treatment costs and improves quality of life.

The first step is identifying which patients would benefit from neoadjuvant therapy, and relatively accurate clinical staging is a prerequisite. Currently, the most commonly used and relatively accurate method is pelvic-enhanced magnetic resonance imaging (MRI), with accuracies of 83.9% for T-staging and 63%-87% for N-staging of rectal tumors (8). However, not every individual benefits from neoadjuvant therapy. Relevant literature indicates that 20-30% of patients show no response to nCRT, and approximately 15% experience disease progression during neoadjuvant treatment (9). Neoadjuvant therapy can also cause difficulties in surgical plane identification, fistula formation, increased mucus, and other side effects. Therefore, although neoadjuvant therapy is generally a favorable choice for most LARC patients, other indicators need to be considered to select those who would truly benefit from neoadjuvant therapy, achieving the goal of precision medicine and developing individualized treatment plans for each patient.

Based on these considerations, this study aims to identify the treatment population that is more sensitive to survival prognosis by retrospectively studying the clinical data characteristics of patients who underwent neoadjuvant therapy in our hospital from 2009 to 2020.

## 2 Materials and methods

### 2.1 Clinical data

This study retrospectively collected data from 284 patients diagnosed with locally advanced rectal cancer (LARC) at the Fourth Hospital of Hebei Medical University between January 2009 and December 2020. These patients were determined to be stage II or stage III (based on the UICC/AJCC TNM staging system) through preoperative high-resolution pelvic magnetic resonance imaging. Among them, 270 patients met the inclusion and exclusion criteria, and their clinical data were compiled before and after neoadjuvant treatment, as well as after surgery. The compiled clinical data included gender, age, comorbidities (such as diabetes, hypertension, cardiovascular diseases), family history, smoking and alcohol history, tumor distance from the anal verge, tumor size, tumor invasion of the bowel wall, cT stage, cN stage, pre-neoadjuvant chemoradiotherapy white blood cell count, neutrophil count, lymphocyte count, platelet count, albumin level, carcinoembryonic antigen (CEA), CA19-9, CA72-4, tumor regression grade (TRG), KRAS gene mutation status, BRAF gene mutation status, and mismatch repair protein expression (MLH1, MSH2, MSH6, PMS2, defined as “microsatellite instability” if there is any protein loss). Additionally, neutrophil-to-lymphocyte ratio (NLR), systemic immune-inflammation index (SII), platelet-to-lymphocyte ratio (PLR), and prognostic nutritional index (PNI) were calculated. Patients with distant metastasis and those who did not undergo curative surgical treatment were excluded from the analysis. All included patients underwent standardized neoadjuvant treatment (DT45.0-50.4 Gy, 1.8-2.0 Gy per fraction, 5 fractions per week, totaling 25–28 fractions) along with 5-fluorouracil or capecitabine chemotherapy. Subsequently, they underwent total mesorectal excision (TME) surgery 6–10 weeks after completing neoadjuvant treatment. Postoperative follow-up was conducted until October 2022, and the data were compiled in December 2022. The follow-up endpoints were survival outcomes, disease-free survival (DFS), and overall survival (OS), and statistical analysis was performed to identify relevant influencing factors.

### 2.2 Inclusion and exclusion criteria

#### 2.2.1 Inclusion criteria

Pathologically confirmed rectal adenocarcinoma, including special types of adenocarcinoma.
All patients underwent digital rectal examination, colonoscopy, enhanced pelvic MRI, chest CT, and other examinations before neoadjuvant therapy, confirming that the tumor distance from the anal margin was not greater than 10 cm.
Clinical staging based on imaging indicated stage II or III locally advanced rectal cancer without distant metastasis.
Completion of standardized neoadjuvant therapy and TME surgery.
Complete clinical case data of the patients.

#### 2.2.2 Exclusion criteria

Patients with contraindications to neoadjuvant therapy or surgery.
Patients who developed metastasis, obstruction, perforation, or bleeding and required emergency surgery before or during treatment.
Patients with hereditary colorectal cancer, such as Lynch syndrome or familial adenomatous polyposis.
Patients with inflammatory bowel disease, such as Crohn’s disease or ulcerative colitis.
Patients with concomitant malignancies in other sites or multiple colorectal cancers.
Loss to follow-up.

### 2.3 Study methods

Retrospective analysis was conducted on the clinical case data of 270 LARC patients who met the inclusion and exclusion criteria. The observed endpoints were DFS and OS. The correlation between various factors and survival prognosis was analyzed.

### 2.4 Observational indicators and evaluation criteria

#### 2.4.1 Follow-up

The Hebei Medical University Fourth Hospital medical records management system was used for follow-up, which included retrospective medical record review, clinic visits, and telephone interviews. The follow-up included survival outcomes, disease progression time, total survival time, and the endpoint event was death due to rectal tumor. All patients were followed up until October 2022.

### 2.5 Statistical methods

IBM SPSS Statistics 23 software (IBM Corp., New York, USA) was used for statistical analysis. Two independent sample t-tests were used for quantitative data with two groups to analyze intergroup differences. The equality of variances was determined based on the results of the Levene’s test. If the p-value > 0.05, the variances were equal, and a t-test was directly used. Otherwise, if the variances were unequal, the corresponding corrected t-value results (adjusted t-test) were chosen. One-way analysis of variance was used to analyze intergroup differences for quantitative data with three or more groups. Factors with statistical significance (p <0.05) in the univariate analysis were included in the multivariate analysis using logistic regression.

## 3 Results

### 3.1 Patients

This study retrospectively collected data from 270 patients diagnosed with LARC. General Patient Information The characteristics of the 270 patients were shown in **Table 1**.

**Table 1:** Subgroups of 270 LARC patients receiving neoadjuvant chemoradiotherapy (nCRT).

Clinical and Pathological Factors	Group	Number of Cases (%)
Gender	Male	172 (63.7)
	Female	98 (36.3)
Age (years)	>65	67 (24.8)
	≤65	203 (75.2)
Underlying Diseases	Present	111 (41.1)
	Absent	159 (58.9)
Smoking	Yes	105 (38.9)
	No	165 (61.1)
Alcohol Consumption	Yes	89 (33.0)
	No	181 (67.0)
Family History	Present	18 (6.7)
	Absent	252 (93.3)
CEA(ng/mL)	>5	184(68.1)
	≤5	86 (31.9)
CA19-9(U/mL)	>30	126 (46.7)
	≤30	144 (53.3)
CA72-4(U/mL)	>7	122 (45.2)
	≤7	148 (54.8)
White Blood Cell Count(×109/L)	>6.12	165 (61.1)
	≤6.12	105 (38.9)
Neutrophil Count(×109/L)	>4.11	166 (61.5)
	≤4.11	104 (38.5)
Lymphocyte Count(×109/L)	>1.50	164 (60.7)
	≤1.50	106 (39.3)
Platelet Count(×109/L)	Normal	142 (52.6)
	Abnormal	128 (47.4)
Albumin(g/l)	Normal	199 (73.7)
	Abnormal	71 (26.3)
Tumor Distance from Anal Margin(cm)	>5	210 (77.8)
	≤5	60 (22.2)
NLR	>2.64	136 (50.4)
	≤2.64	134 (49.6)
PLR	>61.46	135 (50.0)
	≤61.46	135 (50.0)
SII	>683.16	135(50.0)
	≤683.16	135 (50.0)
PNI	>49.23	135 (50.0)
	≤49.23	135 (50.0)
Tumor Involvement of Bowel	>50	218 (80.7)
	≤50	52 (19.3)
cT Stage	T1-2	16 (5.9)
	T3	170 (63.0)
	T4	84 (31.1)
cN Stage	N0	15 (5.6)
	N+	255 (94.4)
TRG Grade	0	34 (12.6)
	1-2	151 (55.9)
	3	85 (31.5)
KRAS Mutation	Present	19 (7.0)
	Absent	251 (93.0)
BRAF Mutation	Present	5 (1.9)
	Absent	265 (98.1)
Microsatellite Status	Unstable	20 (7.4)
	Stable	250 (92.6)
Outcomes (As of October 2022)	Survived	190 (70.4)
	Deceased	80 (29.6)

The median disease-free survival (DFS) was 39.8 months, with an average DFS of 49 months. The median overall survival (OS) was 42.4 months, with an average OS of 52.6 months. As of October 2022, there were 190 cases (70.4%) who were still alive and 80 cases (29.6%) who had died. See **Table 1** for details.

### 3.2 Univariate analysis results

The univariate analysis shows that patient age, CEA level, lymphocyte count, albumin, NLR, SII, PNI, tumor distance from the anus, tumor size, tumor invasion of the intestinal wall, cT stage, cN stage, TRG grade, KRAS status, and microsatellite status are associated with survival (**Table 2**).

**Table 2:** Univariate analysis of various factors affecting neoadjuvant prognosis.

Factor	Classification	DFS (month)			P-value	OS (month)			P-value
		Mean value	Number of Cases	Standard Deviation		Average Value	Number of Cases	Standard Deviation	
gender	Male	47.163	172	29.037	0.234	50.753	172	27.153	0.202
	Female	52.142	98	34.949		55.760	98	32.834	
The age is No <65 years old	Yes	51.502	203	31.355	0.021	55.002	203	29.367	0.018
	No	41.300	67	30.244		45.204	67	28.382	
Underlying Diseases	Yes	47.526	111	30.813	0.528	51.132	111	28.856	0.503
	No	49.979	159	31.760		53.575	159	29.791	
Smoking	Yes	50.659	105	32.046	0.481	54.059	105	30.246	0.508
	No	47.896	165	30.932		51.623	165	28.870	
Alcohol Consumption	Yes	48.604	89	30.287	0.893	51.487	89	28.710	0.672
	No	49.151	181	31.925		53.103	181	29.769	
Family History	Yes	55.942	16	34.193	0.158	56.898	16	33.562	0.274
	No	48.688	253	31.133		52.457	253	29.098	
CEA ≤ 5ng/ml	Yes	54.687	91	32.406	0.032	58.149	91	30.294	0.026
	No	46.064	179	30.465		49.734	179	28.575	
CA19-9 ≤ 30U/ml	Yes	49.329	144	31.544	0.841	53.566	144	29.055	0.553
	No	48.561	126	31.224		51.433	126	29.825	
CA724 ≤ 7U/ml	Yes	50.875	148	31.535	0.272	54.895	148	29.007	0.152
	No	46.660	122	31.073		49.750	122	29.702	
White Blood Cell Count ≤ 6.12	Yes	49.934	166	32.230	0.524	53.555	166	30.420	0.488
	No	47.433	104	29.951		50.999	104	27.713	
Neutrophil Count ≤ 4.11	Yes	49.071	167	31.235	0.947	52.785	167	29.459	0.879
	No	48.807	103	31.660		52.223	103	29.395	
Lymphocyte Count ≤ 1.50	Yes	44.701	104	27.660	0.065	48.271	104	25.698	0.047
	No	51.645	166	33.241		55.264	166	31.243	
Lymphocyte Count	Yes	51.857	128	33.212	0.151	54.300	128	31.813	0.363
	No	46.368	142	29.426		51.011	142	27.021	
Albumin<40g/l	Yes	40.447	71	26.062	0.003	44.081	71	23.782	0.001
	No	52.011	199	32.540		55.599	199	30.623	
NLR ≤ 2.64	Yes	55.392	136	33.717	0.001	58.893	136	31.558	<0.001
	No	42.453	134	27.341		46.154	134	25.548	
PLR ≤ 61.46	Yes	49.763	135	30.691	0.678	52.583	135	29.190	0.995
	No	48.177	135	32.070		52.558	135	29.679	
SII ≤ 683.16	Yes	52.637	135	32.704	0.054	56.705	135	30.507	0.02
	No	45.303	135	29.581		48.436	135	27.709	
PNI ≤ 49.23	Yes	44.714	135	27.864	0.025	48.354	135	25.810	0.018
	No	53.226	135	34.040		56.787	135	32.108	
Tumor Distance from Anal Margin ≤ 5cm	Yes	38.466	60	26.444	0.001	44.659	60	23.969	0.008
	No	51.972	210	32.031		54.831	210	30.426	
Tumor length ≤ 5cm	Yes	53.600	113	34.766	0.046	57.984	113	32.007	0.012
	No	45.638	157	28.269		48.674	157	26.767	
Tumor Involvement of Bowel ≤ 50%	Yes	65.424	52	40.811	0.001	70.671	52	36.166	<0.001
	No	45.046	218	27.308		48.253	218	25.796	
cT Stage	1-2	85.054	16	42.709	<0.001	88.417	16	38.996	<0.001
	3	50.592	170	30.762		53.948	170	28.811	
	4	38.815	84	23.790		42.954	84	22.080	
cN Stage	N0	73.978	15	46.008	0.044	82.849	15	37.036	0.005
	N+	47.499	255	29.739		50.789	255	27.951	
TRG Grade	0	70.733	34	35.977	<0.001	71.864	34	35.309	<0.001
	1-2	52.984	151	31.537		56.202	151	29.614	
	3	33.135	85	19.655		38.402	85	18.195	
KRAS Mutation	Yes	49.857	251	32.081	0.004	53.569	251	30.022	<0.001
	No	37.254	19	15.100		39.382	19	13.588	
BRAF Mutation	Yes	33.707	5	22.331	0.272	35.780	5	21.111	0.198
	No	49.258	265	31.444		52.887	265	29.451	
BRAF Mutation	Yes	46.597	250	28.764	0.005	49.983	250	26.967	0.001
	No	78.643	20	45.400		84.915	20	38.757	

#### 3.2.1 Age

The average age was 57.9 years, with a median age of 59 years. Patients were divided into two groups based on whether their age was below or equal to 65 years. There were 203 patients (75.2%) below 65 years of age. The group with older patients had an average DFS and OS of 41.3 months and 45.2 months, respectively. The group with younger patients had an average DFS and OS of 51.6 months and 55.0 months, respectively. These differences were statistically significant.

#### 3.2.2 CEA

Among the 270 patients, 184 (68.1%) had CEA levels greater than 5 ng/mL, while 86 (31.9%) had levels less than or equal to 5 ng/mL. The group with higher CEA levels had an average DFS and OS of 46.0 months and 49.7 months, respectively. The group with lower CEA levels had an average DFS and OS of 54.7 months and 58.1 months, respectively. These differences were statistically significant (p-values were 0.032 and 0.026, respectively).

Lymphocyte Count: The average lymphocyte count was 1.50 × 10^9/L. Among the patients, 164 (60.7%) had counts greater than 1.50 × 10^9/L, while 106 (39.3%) had counts less than or equal to 1.50 × 10^9/L. The group with higher lymphocyte counts had an average DFS and OS of 51.6 months and 55.3 months, respectively. The group with lower lymphocyte counts had an average DFS and OS of 44.7 months and 48.3 months, respectively. DFS showed no significant difference (p=0.065), while OS showed a significant difference (p=0.047).

#### 3.2.3 Albumin

Among the 270 patients, 199 (73.7%) had normal albumin levels, while 71 (26.3%) had lower levels. The group with normal albumin levels had an average DFS and OS of 52.0 months and 55.6 months, respectively. The group with lower albumin levels had an average DFS and OS of 40.4 months and 44.1 months, respectively. These differences were statistically significant (p-values were 0.003 and 0.001, respectively).

#### 3.2.4 NLR

The median NLR was 2.64. Based on the median, the population was divided into two groups. The group with higher NLR values had an average DFS and OS of 42.5 months and 46.2 months, respectively. The group with lower NLR values had an average DFS and OS of 55.4 months and 58.9 months, respectively. These differences were statistically significant (p-values were 0.001 and <0.001, respectively).

#### 3.2.5 SII

The median SII was 683.16. Based on the median, the population was divided into two groups. The group with higher SII values had an average DFS and OS of 45.3 months and 48.4 months, respectively. The group with lower SII values had an average DFS and OS of 52.6 months and 56.7 months, respectively. DFS showed no significant difference (p=0.054), while OS showed a significant difference (p=0.020).

PNI: The median PNI was 49.23. Based on the median, the population was divided into two groups. The group with higher PNI values had an average DFS and OS of 53.2 months and 56.8 months, respectively. The group with lower PNI values had an average DFS and OS of 44.7 months and 48.4 months, respectively. These differences were statistically significant (p-values were 0.025 and 0.018, respectively).

#### 3.2.6 Tumor distance from the anus

Among the 270 patients, 210 (77.8%) had tumors located more than 5 cm from the anus, while 60 (22.2%) had tumors located 5 cm or less from the anus. The group with greater tumor distance had an average DFS and OS of 52.0 months and 54.8 months, respectively. The group with shorter tumor distance had an average DFS and OS of 38.5 months and 44.7 months, respectively. These differences were statistically significant (p-values were 0.001 and 0.008, respectively).

#### 3.2.7 Tumor size

Among the 270 patients, 157 (58.1%) had tumor sizes greater than 5 cm, while 113 (41.9%) had tumor sizes 5 cm or less. The group with larger tumors had an average DFS and OS of 45.6 months and 48.7 months, respectively. The group with smaller tumors had an average DFS and OS of 53.6 months and 58.0 months, respectively. These differences were statistically significant (p-values were 0.046 and 0.012, respectively).

#### 3.2.8 Tumor invasion of the intestinal wall

Among the 270 patients, 218 (80.7%) had tumors that invaded more than 50% of the intestinal wall, while 52 (19.3%) had tumors that invaded 50% or less. The group with more extensive invasion had an average DFS and OS of 45.0 months and 48.3 months, respectively. The group with less extensive invasion had an average DFS and OS of 65.4 months and 70.7 months, respectively. These differences were statistically significant (p-values were 0.001 and <0.001, respectively).

#### 3.2.9 cT stage

Among the patients, 16 (5.9%) were in stage 1-2, 170 (63.0%) were in stage T3, and 84 (31.1%) were in stage T4. The average DFS and OS for patients in stage 1–2 were 85.1 months and 88.4 months, respectively. For patients in stage T3, the average DFS and OS were 50.6 months and 54.0 months, respectively. For patients in stage T4, the average DFS and OS were 49.0 months and 52.6 months, respectively. These differences were statistically significant (p-values were all <0.001).

#### 3.2.10 cN stage

Among the patients, 15 (5.6%) were in stage N0, while 255 (94.4%) were in stage N+. The average DFS and OS for patients in stage N0 were 74.0 months and 82.8 months, respectively. For patients in stage N+, the average DFS and OS were 47.5 months and 50.8 months, respectively. These differences were statistically significant (p-values were 0.044 and 0.005, respectively).

#### 3.2.11 TRG grade

Among the patients, 34 (12.6%) were in grade 0, 151 (55.9%) were in grade 1-2, and 85 (31.5%) were in grade 3. The average DFS and OS for patients in grade 0 were 70.7 months and 71.9 months, respectively. For patients in grade 1-2, the average DFS and OS were 53.0 months and 56.2 months, respectively. For patients in grade 3, the average DFS and OS were 33.1 months and 38.4 months, respectively. These differences were statistically significant (p-values were all <0.001).

#### 3.2.12 KRAS status

Among the 270 patients, 19 (7%) had KRAS mutations, while 251 (93%) did not. The group with KRAS mutations had an average DFS and OS of 37.3 months and 39.4 months, respectively. The group without KRAS mutations had an average DFS and OS of 49.9 months and 53.6 months, respectively. These differences were statistically significant (p-values were 0.004 and <0.001, respectively).

#### 3.2.13 Microsatellite status

Among the 270 patients, 20 (7.4%) had microsatellite instability, while 250 (92.6%) had microsatellite stability. The group with microsatellite instability had an average DFS and OS of 78.6 months and 84.9 months, respectively. The group with microsatellite stability had an average DFS and OS of 46.6 months and 50.0 months, respectively. These differences were statistically significant (p-values were 0.005 and 0.001, respectively). (Refer to **Table 2** for details).

### 3.3 Results of multivariable analysis

The indices with P<0.05 from the univariate analysis were included in the multivariable analysis. DFS and OS were grouped based on whether they reached 36months, and binary logistic regression analysis was used for the multivariable analysis. The results, as shown in **Table 3**, indicated that age (=0.385, P=0.007), NLR (HR=0.294, P=0.011), cT stage (HR=0.287, P=0), and TRG grade (HR=0.273, P=0) were significant factors affecting DFS in nCRT patients. Specifically, patients under the age of 65, with NLR lower than the median level, lower cT stage, and lower TRG grade, had longer DFS. Regarding OS, age (HR=0.497, P=0.035), cT stage (HR=0.387, P=0.001), and TRG grade (HR=0.307, P=0) were significant factors affecting OS in nCRT patients (**Table 4**).

**Table 3:** Multi-factor analysis (DFS regression results).

Factor	B	Standard Error	Wald	P	EXP(B)	EXP(B)95%CI	
						Lower	Upper
Age≥65	-0.955	0.353	7.316	0.007	0.385	0.193	0.769
CEA>5ng/ml	-0.022	0.364	0.003	0.953	0.979	0.48	1.996
Lymphocyte>1.50×109/L	0.174	0.362	0.232	0.63	1.19	0.586	2.418
Normal Albumin	0.525	0.395	1.769	0.184	1.691	0.78	3.665
NLR>2.64	-1.223	0.479	6.527	0.011	0.294	0.115	0.752
SII>683.16	0.498	0.479	1.081	0.299	1.645	0.643	4.207
PNI>49.23	-0.134	0.362	0.138	0.711	0.874	0.43	1.778
Tumor-Anal Distance>5cm	0.645	0.386	2.788	0.095	1.905	0.894	4.061
Tumor Length>5cm	0.639	0.362	3.12	0.077	1.894	0.932	3.848
Invasion of Intestinal Wall>50%	-0.549	0.466	1.392	0.238	0.577	0.232	1.438
cT Stage	-1.247	0.306	16.651	<0.001	0.287	0.158	0.523
cN Stage	0.372	0.753	0.245	0.621	1.451	0.332	6.349
TRG Grade	-1.299	0.335	14.987	<0.001	0.273	0.141	0.527
KRAS Mutation	0.196	0.552	0.126	0.722	1.216	0.413	3.586
Microsatellite Status	0.661	0.687	0.926	0.336	1.937	0.504	7.451

**Table 4:** Multi-factor analysis (OS regression results).

Factor	B	Standard Error	Wald	P	EXP(B)	EXP(B)95%CI	
						Lower	Upper
Age≥65	-0.7	0.332	4.437	0.035	0.497	0.259	0.953
CEA>5ng/ml	-0.125	0.341	0.135	0.714	0.882	0.452	1.723
Lymphocyte>1.50×109/L	0.242	0.337	0.513	0.474	1.273	0.657	2.466
Normal Albumin	0.617	0.38	2.638	0.104	1.854	0.88	3.905
NLR>2.64	-0.819	0.456	3.23	0.072	0.441	0.18	1.077
SII>683.16	0.285	0.436	0.426	0.514	1.329	0.565	3.126
PNI>49.23	-0.145	0.352	0.169	0.681	0.865	0.434	1.724
Tumor-Anal Distance>5cm	0.37	0.364	1.033	0.309	1.448	0.709	2.958
Tumor Length>5cm	0.534	0.34	2.463	0.117	1.706	0.876	3.323
Invasion of Intestinal Wall>50%	-0.518	0.454	1.303	0.254	0.596	0.245	1.45
cT Stage	-0.948	0.291	10.61	0.001	0.387	0.219	0.685
cN Stage	-0.716	0.819	0.764	0.382	0.489	0.098	2.433
TRG Grade	-1.18	0.319	13.732	<0.001	0.307	0.164	0.573
KRAS Mutation	0.206	0.536	0.147	0.701	1.228	0.43	3.511
Microsatellite Status	0.914	0.731	1.566	0.211	2.495	0.596	10.448

## 4 Discussion

As the incidence and mortality rates of colorectal cancer continue to rise in China, especially with a high proportion of rectal cancer patients, early symptoms of rectal cancer lack specificity and are mostly manifested as rectal bleeding and changes in bowel habits. Many patients do not pay attention to these symptoms, and by the time they seek medical attention, most tumors have already entered the locally advanced stage. For patients with locally advanced rectal cancer, neoadjuvant therapy is crucial, as its effectiveness directly affects patient prognosis. Therefore, it is important to identify patients who are sensitive to neoadjuvant therapy, which is also a requirement for individualized treatment and precision medicine.

This study confirms that patient gender, underlying diseases, smoking and alcohol history, family history, white blood cell count, neutrophil count, platelet count, CA19-9, CA72-4, PLR, and BRAF status are not significantly associated with prognosis. These indicators are currently controversial, with some studies reporting that female patients, those with a history of smoking and alcohol consumption, high platelet count, and CA19–9 levels above the normal range have lower effectiveness of neoadjuvant chemoradiotherapy for locally advanced rectal cancer (10). There are reports that BRAF mutations are associated with resistance to neoadjuvant chemoradiotherapy, but the incidence of BRAF mutations is low, and in this study (11), there were only five cases, which may introduce errors.

The impact of age on neoadjuvant chemoradiotherapy is currently inconclusive, but in this study, both univariate and multivariate analyses found that age is also an indicator that affects survival prognosis. Patients aged ≥65 years had worse disease-free survival (DFS) and overall survival (OS) than younger patients (p-values of 0.021 and 0.018, respectively). The reason for this result may be that the older patients included in the study had a later clinical stage, which may be related to delayed medical treatment and fewer medical examinations (12). Carcinoembryonic antigen (CEA) has been proven by multiple studies to be an important indicator for predicting the effectiveness of neoadjuvant chemoradiotherapy and patient prognosis. This study used CEA >5ng/ml as the grouping criterion and found that patients with CEA ≤5ng/ml had better DFS and OS than those with higher CEA levels, which was statistically significant. However, this study did not find any correlation between other colorectal cancer markers such as CA19–9 and CA72–4 and patient prognosis. Lymphocytes play an important role in tumor immune system, and the main anti-tumor lymphocytes in peripheral blood include B cells, T cells and NK cells. CD8+ cytotoxic T cells are able to recognize and bind to antigens associated with MHC I molecules, which are critical in the adaptive immune response against cancer; CD4+T cells, as auxiliary or regulatory T cells, target or eliminate tumor cells through a variety of ways, and their functions are complex and variable. Studies have shown that rectal cancer patients with lymphocyte ratio in peripheral blood (that is, the proportion of lymphocytes in white blood cells per unit volume of blood) ≥20% can show significant improvement in pathological response after receiving neoadjuvant therapy (13). However, the study also noted that lymphocytopenia has an important impact on patient outcomes. Studies have shown that lymphocytopenia weakens immune function, increases the risk of infection, and is closely associated with the risk of tumor recurrence (14, 15). In addition, studies have shown that lymphocytopenia may be associated with reduced long-term survival and treatment outcomes (16–18). Based on the above findings, this study believes that maintaining lymphocyte levels is essential to improve patients’ immune function, improve treatment effect and improve prognosis (19). At the same time, studies should focus on co-existing effects with other cancer types (such as anal cancer) in terms of hematotoxicity or lymphocytopenia, and on this basis, comparison and analysis should be conducted to fully understand the complexity of this issue. To further optimize the treatment regimen, this study recommends timely detection and intervention through individualized treatment, adjustment of the intensity of chemotherapy and radiotherapy, and regular monitoring of patients’ blood indicators, especially lymphocyte counts. In addition, immunity can be enhanced by providing adequate nutritional support, especially foods rich in vitamins and minerals; At the same time, infection prevention, such as vaccination, the use of antibiotics and other measures to reduce the negative impact of lymphocytopenia. This study found that patients with higher NLR (neutrophil to lymphocyte ratio) did not differ significantly in disease-free survival, but overall survival was longer (p =0.065 and 0.047). For the pCR (non-progressive recurrence) group with high NLR, the difference in NLR values was statistically significant compared with other groups (20). Although NLR had limited predictive value for pathological complete response (pCR), the difference in NLR between pCR and non-PCR groups was significant [p=0.035] (21). In general, higher lymphocyte levels contribute to improved immune function and prognosis in patients, while measures such as individualized treatment and nutritional support can effectively reduce the impact of lymphocytopenia.

There have been many studies on the distance of the tumor from the anal verge, but the conclusions are not consistent. Lower rectal cancer with a distance ≤5cm has a higher pCR rate (22). This may be because the position of the upper rectal colon is less fixed than the lower rectum, and the radiation dose is reduced due to the obstruction of pelvic organs such as the bladder and uterus. A more detailed study divided the distance in 2cm increments from 4cm and found that tumors with a distance from the anal verge <4cm or >8cm were more difficult to achieve pCR (23). However, some studies have indicated that the distance of the tumor from the anal verge is not related to pCR (24). The conclusion drawn from this study is that patients with a distance of the tumor from the anal verge >5cm had better DFS and OS compared to patients with a distance ≤5cm (both p-values <0.05), which is consistent with current mainstream research. However, in a meta-analysis of 1913 patients, there were no differences in tumor size and distance from the tumor to the anus between patients with and without pCR (6). Felice’s study found that a tumor length >5cm measured by colonoscopy before neoadjuvant chemoradiotherapy often indicates a poor response to treatment (25), and this study also confirmed that tumor length is correlated with patient prognosis, with patients with a tumor length >5cm having a worse prognosis. Multiple studies have shown that the involvement of bowel circumference by the tumor is related to pCR (26), but the specific values are not yet unified. Some researchers have stated that patients with involvement of <1/3 of the bowel circumference are more likely to achieve pCR. Das et al. showed that a tumor-to-bowel circumference ratio <40% is associated with a high rate of pCR. This study confirmed that a tumor-to-bowel circumference ratio ≤50% is associated with a better prognosis, which is statistically significant. Multiple studies have demonstrated that pre-treatment T stage and N stage are closely related to pCR status (27). The higher the cT stage, the deeper the tumor infiltration. Patients with a higher T stage have a significantly worse response to treatment and can predict pCR (28). A study including 23,747 LARC patients in other countries showed that as the cT1-cT4 and cN0-cN2 stages change, the pCR rate gradually decreases, and prognosis worsens accordingly. Another study demonstrated a significant association between cT stage and 5-year DFS and 5-year OS in LARC patients who received neoadjuvant chemoradiotherapy (29), while cN stage was not associated with 5-year DFS and 5-year OS. This study confirmed that cT stage, cN stage, tumor regression grade (TRG), and achievement of pCR status are significantly correlated with DFS and OS. Tumor stage and the regression response after neoadjuvant therapy indicate the timing of tumor development and sensitivity to neoadjuvant treatment. Patients with higher T stage and N stage have a higher tumor burden, and a larger tumor burden leads to more severe hypoxia in the tumor microenvironment, which may promote cancer cell progression and increase resistance to neoadjuvant treatment (30).

In terms of gene mutations, studies have found that individuals who respond poorly to neoadjuvant chemoradiotherapy are more likely to have KRAS mutations (31), and microsatellite instability (MSI) patients are more likely to achieve pCR compared to microsatellite stable patients (32). BRAF gene mutations have been found to be associated with resistance to neoadjuvant chemoradiotherapy in LARC patients (33). This study found that patients with KRAS mutations had worse prognosis. Among the 270 patients included in the study, there were 19 cases of KRAS mutation, which is lower than the reported mutation rate, possibly due to insufficient sample size, so the supporting evidence is not sufficient. The study did not find a significant correlation between BRAF status and prognosis, but MSI status showed significant differences in both DFS and OS, with microsatellite stable patients having a worse prognosis.

Some studies have also explored the correlation between the occurrence of mucin pools (colloid reactions) in postoperative pathology and prognosis in patients who received neoadjuvant therapy. A considerable proportion of rectal cancer patients who received neoadjuvant therapy have mucin pools, which can be further divided into pools with and without cells. Some researchers have reported that the likelihood of mucin pools without cells occurring in all LARC patients after neoadjuvant chemoradiotherapy is approximately 12% to 35%. Among patients who achieved pCR, 27% of patients had mucin pools without cells (34). This study also indicated that patients with mucin pools had better prognosis, but residual tumor cells in mucin pools were associated with poor prognosis. Another meta-analysis described the formation of mucin pools in 1,947 patients and found that the presence of mucin pools was not associated with gender, T stage, N stage, tumor regression, complete pathological response rate, lymphatic vessel invasion, perineural invasion, differentiation, margin status, local or distant recurrence, disease-free survival, or overall survival. There are still many potential factors that can affect patient prognosis after neoadjuvant therapy (35).

On average, postoperative pathology after neoadjuvant treatment reveals a mean reduction of 3.9 lymph nodes, and dissecting at least 12 lymph nodes can improve OS and DFS in patients (36). Other factors such as gut microbiota, transcription factors, non-coding RNA, chemokines, etc., can also have an impact on neoadjuvant therapy effectiveness and patient prognosis.

Deep learning has shown great potential in the diagnosis of colorectal cancer (CRC), particularly in areas such as histopathological image analysis, tumor detection and segmentation, imaging assistance, and prognosis prediction (37). Firstly, deep learning algorithms, especially convolutional neural networks (CNNs), can automatically analyze histopathological images of colorectal cancer, identifying subtle differences between cancerous and normal cells. By learning from a large amount of image data, these models can extract cancer-specific features, thereby improving the accuracy of image classification. In tumor detection and segmentation, deep learning can accurately identify and segment tumor regions, enhancing the sensitivity of early detection and helping pathologists reduce human errors (38). Secondly, in imaging studies, deep learning technologies are applied to process CT, MRI, ultrasound, and other imaging data, helping to detect subtle lesions and early tumor markers, improving the accuracy and efficiency of diagnostic imaging. This is especially important in resource-limited environments, where rapid and efficient screening can be achieved (39). Moreover, deep learning also plays an important role in the prognosis evaluation and personalized treatment of colorectal cancer. By integrating clinical data, pathological images, and imaging data, deep learning models can predict tumor aggressiveness, metastasis risk, and patient response to different treatments, supporting the development of personalized treatment plans. This data-driven approach can improve patient survival rates and enhance treatment outcomes. Deep learning can also assist doctors in managing large volumes of data, significantly improving diagnostic efficiency and reducing diagnostic time and resource consumption, which is crucial for large-scale screening and accelerating diagnosis (40, 41). In conclusion, deep learning provides strong support across multiple stages of colorectal cancer, including early detection, tumor segmentation, imaging analysis, prognosis prediction, and personalized treatment. It holds promise to further enhance the accuracy, efficiency, and treatment outcomes of colorectal cancer diagnosis in the future. As technology continues to advance, deep learning will play an increasingly important role in the clinical application of colorectal cancer, driving cancer diagnosis and treatment toward more precise and efficient directions.

Postoperative complications in colorectal cancer surgery may include infection, bleeding, intestinal dysfunction, anastomotic leak, pulmonary complications, and liver dysfunction. These complications not only affect patient recovery but may also increase hospital stay and treatment costs. Infection is typically the most common complication, especially at the surgical site and within the abdominal cavity (42). Anastomotic leak is a severe complication in colorectal cancer surgery, which can lead to peritonitis or sepsis (43). Postoperative intestinal dysfunction and ileus may affect patient recovery (44), and pulmonary complications such as pneumonia are also common, especially in elderly patients or those with underlying conditions (45). Management of postoperative complications requires a comprehensive consideration of the patient’s baseline health status, type of surgery, and postoperative care. Early identification and intervention can help reduce the occurrence of these complications and improve prognosis. Butyrylcholinesterase (BuChE), as a predictive biomarker for postoperative complications, has significant clinical application potential. Its level changes are closely related to various factors such as inflammatory response, liver function damage, anesthetic drug metabolism, and organ failure. Studies have shown that abnormal changes in BuChE levels can provide early warning for postoperative complications such as infection, liver injury, or multiple organ failure, thus assisting doctors in early intervention and personalized treatment. Combined with other biomarkers, the detection of BuChE is expected to improve the accuracy of complication prediction and optimize postoperative management and recovery (46).

The Internet of Things (IoT) plays an increasingly important role in the diagnosis, treatment, and management of colorectal cancer. Through smart sensors and devices, IoT can monitor patients’ vital signs, postoperative recovery, and potential complications in real time, assisting doctors in providing personalized treatment. For example, wearable devices can monitor patients’ activity levels, body temperature, heart rate, and other factors, providing real-time data on recovery and helping to identify infections or other complications promptly. IoT technology can also be integrated with telemedicine systems to support remote monitoring and management of patients, reducing hospital stay and improving treatment efficiency. Additionally, IoT is applied in colorectal cancer screening, where smart detection devices and data analysis can enhance the accuracy of early screenings, promoting early detection and timely intervention of cancer (47).

This study has several limitations. It is a retrospective study, and many genetic and molecular data at the gene level are not available. Additionally, the study has a large time span, and treatment concepts and levels may have been updated, causing biases.

## 5 Conclusion

patient age, CEA levels, lymphocyte count, albumin, NLR, SII, PNI, tumor distance from the anal verge, tumor length, tumor involvement of the bowel circumference, cT stage, cN stage, TRG grade, KRAS status, and MSI status were correlated with survival. Age, cT stage, NLR, and TRG grade were identified as independent factors affecting the survival prognosis of LARC patients. Specifically, younger age (<65 years), NLR below the median level, lower cT stage, and lower TRG grade were associated with better prognosis.

## Data Availability

The original contributions presented in the study are included in the article/supplementary material. Further inquiries can be directed to the corresponding author.
